# Sequential Dysfunction and Progressive Depletion of *Candida albicans*-Specific CD4 T Cell Response in HIV-1 Infection

**DOI:** 10.1371/journal.ppat.1005663

**Published:** 2016-06-09

**Authors:** Fengliang Liu, Xiuzhen Fan, Sarah Auclair, Monique Ferguson, Jiaren Sun, Lynn Soong, Wei Hou, Robert R. Redfield, Deborah L. Birx, Silvia Ratto-Kim, Merlin L. Robb, Jerome H. Kim, Nelson L. Michael, Haitao Hu

**Affiliations:** 1 Department of Microbiology & Immunology and Sealy Center for Vaccine Development, University of Texas Medical Branch, Galveston, Texas, United States of America; 2 Division of Infectious Diseases, University of Texas Medical Branch, Galveston, Texas, United States of America; 3 School of Basic Medical Sciences, Wuhan University, Wuhan, Hubei, China; 4 Institute of Human Virology and Division of Infectious Diseases, University of Maryland School of Medicine, Baltimore, Maryland, United States of America; 5 U.S. Military HIV Research Program, Water Reed Army Institute of Research, Silver Spring, Maryland, United States of America; 6 U.S. Military HIV Research Program, Henry M. Jackson Foundation, Silver Spring, Maryland, United States of America; 7 International Vaccine Institute, Seoul, Republic of Korea; U.S. Military HIV Research Program, Water Reed Army Institute of Research, Silver Spring, Maryland, United States of America; Vaccine Research Center, UNITED STATES

## Abstract

Loss of immune control over opportunistic infections can occur at different stages of HIV-1 (HIV) disease, among which mucosal candidiasis caused by the fungal pathogen *Candida albicans (C*. *albicans)* is one of the early and common manifestations in HIV-infected human subjects. The underlying immunological basis is not well defined. We have previously shown that compared to cytomegalovirus (CMV)-specific CD4 cells, *C*. *albicans-specific* CD4 T cells are highly permissive to HIV *in vitro*. Here, based on an antiretroviral treatment (ART) naïve HIV infection cohort (RV21), we investigated longitudinally the impact of HIV on *C*. *albicans*- and CMV-specific CD4 T-cell immunity *in vivo*. We found a sequential dysfunction and preferential depletion for *C*. *albicans-*specific CD4 T cell response during progressive HIV infection. Compared to Th1 (IFN-γ, MIP-1β) functional subsets, the Th17 functional subsets (IL-17, IL-22) of *C*. *albicans-*specific CD4 T cells were more permissive to HIV *in vitro* and impaired earlier in HIV-infected subjects. Infection history analysis showed that *C*. *albicans-*specific CD4 T cells were more susceptible to HIV *in vivo*, harboring modestly but significantly higher levels of HIV DNA, than CMV-specific CD4 T cells. Longitudinal analysis of HIV-infected individuals with ongoing CD4 depletion demonstrated that *C*. *albicans-*specific CD4 T-cell response was preferentially and progressively depleted. Taken together, these data suggest a potential mechanism for earlier loss of immune control over mucosal candidiasis in HIV-infected patients and provide new insights into pathogen-specific immune failure in AIDS pathogenesis.

## Author Summary

HIV infection is closely associated with enhanced host susceptibility to various opportunistic infections (OIs), among which mucosal candidiasis caused by the fungal pathogen *Candida albicans* (*C*. *albicans*) is an early and common manifestation. Even in the era of effective ART, mucosal candidiasis is still a clinically relevant presentation in HIV-infected patients. The underlying mechanisms are not well defined. CD4-mediated immunity is the major host defense mechanism against *C*. *albicans*. We here investigated a group of ART naïve, HIV-infected human subjects and examined longitudinally the impact of HIV on *C*. *albicans*-specific CD4 T-cell immunity as compared to CD4 T-cell immunity specific for CMV, another opportunistic pathogen that usually does not cause active disease in early HIV infection. We found that *C*. *albicans*-specific CD4 T cells were more susceptible to HIV *in vivo* and were preferentially depleted in progressive HIV-infected individuals as compared to CMV-specific CD4 T cells. Of importance, we also found that in these HIV-infected subjects *C*. *albicans*-specific CD4 T cell response manifested a sequential dysfunction with earlier impairment of Th17, but not Th1, functions. Our study suggests an immunological basis that helps explain the earlier and more common onsets of mucosal candidiasis in progressive HIV-infected patients.

## Introduction

Untreated HIV infection causes progressive depletion of human CD4 T cells, leading to impaired cellular immunity, enhanced susceptibility to opportunistic infections (OIs) and development of acquired immunodeficiency syndrome (AIDS) [1–3]. Although the loss of immune control over OIs is known to be generally associated with overall reduction in CD4 T cells, HIV cohort studies have found that OI reactivation can occur at different stages of HIV disease and is not strictly associated with total CD4 loss [4–6]. For instance, while the opportunistic pathogen *Mycobacterium tuberculosis* (MTB) can cause active disease relatively early during HIV infection [7], cytomegalovirus (CMV) infection rarely causes evident diseases at early stage [8, 9]. These observations have suggested that host immunity specific for opportunistic pathogens may be impaired or lost at different stages of HIV disease [10–12]. In support, an important study by Geldmacher *et al*. demonstrated that compared to CMV, MTB-specific CD4 T cells are preferentially infected and depleted in HIV-infected human subjects [10, 13].

Mucosal candidiasis, predominantly caused by the commensal fungal organism *Candida albicans (C*. *albicans)*, is one of the most common and earliest manifestations in HIV-infected subjects [14, 15]. In immune competent humans, *C*. *albicans* can be readily detected without overt signs of clinical disease [16]. However, under immune compromised conditions such as in AIDS patients, *C*. *albicans* can quickly cause active infections in multiple tissues, including oral mucosa [17]. Evidence has shown that about 50–90% of HIV-infected individuals could manifest an episode of oral candidiasis during their progression to AIDS [18, 19]. Even with the introduction of potent antiretroviral treatment (ART), oropharyngeal and esophageal candidiasis are still the two clinically relevant presentations in HIV-infected patients [20]. The underlying immunological basis for early and profound onsets of pathogenic *C*. *albicans* infections in HIV-infected individuals is not fully defined.

*C*. *albicans* exposure induces strong cellular immunity, as evidenced by the skin-test reactivity and *in vitro* lymphocyte proliferative response [21, 22]. Majority of evidence obtained so far from animal models and human studies has suggested CD4-mediated cellular immunity as the predominant host defense mechanism against *C*. *albicans* infection [23–30], although involvement of specific functional facets of CD4 T-cell immunity, for instance, Th1 vs. Th17 response, has been obscure. It was initially suggested that Th1 response was the key mediator of immunity [31]. More recently, increasing evidence has indicated that Th17, but not Th1, response is critical for immune protection against mucosal candidiasis [25, 32, 33]. Importantly, in the setting of HIV infection, limited information is currently available regarding the longitudinal impact of HIV on different functional facets of anti-*C*. *albicans* CD4 T-cell immunity in HIV-infected individuals.

To explore the effect of HIV on different antigen-specific CD4 T cells, we have previously described an *in vitro* system, where HIV susceptibility and the associated phenotypes of antigen-specific CD4 cells can be examined [12, 34]. We have found that human *C*. *albicans-*specific CD4 T cells are highly permissive to HIV infection *in vitro* compared to CMV-specific CD4 T cells [12]. It remains to be determined as to how HIV affects these two groups of pathogen-specific CD4 T-cell immunity *in vivo* in HIV-infected subjects. RV21 is an antiretroviral treatment (ART) naïve, longitudinal HIV-infection cohort established by the U.S. Military HIV Research (MHRP) and the HIV-infected subjects enrolled in this cohort were followed up for 2 to 6 years. In the current study, we studied HIV-infected subjects in the RV21 cohort who manifested ongoing CD4 depletion. Using PBMC samples from these individuals, we comparatively examined the longitudinal impact of HIV on functional profiles and magnitudes of *C*. *albicans-* and CMV-specific CD4 T cell responses *in vivo* during HIV disease progression. Our data showed that there was a sequential dysfunction for *C*. *albicans-*specific CD4 T cell response with an earlier and more profound impairment of Th17-associated functions (IL-17, IL-22) in HIV infection. Further analyses identified that compared to CMV-specific CD4 T cells, *C*. *albicans-*specific CD4 T cells were more susceptible to HIV *in vivo* and preferentially depleted in these HIV-infected subjects.

## Results

### *C*. *albicans*-specific CD4 T cells manifest distinct functional characteristics from CMV-specific CD4 T cells

Antigen-specific T cell responses elicited by different pathogens can be qualitatively distinct. In our previous studies [12, 34], we have reported an *in vitro* system for examining the susceptibility of antigen-specific human CD4 T cells to HIV infection and the associated phenotypic and functional characteristics (Fig A in S1 Appendix). We here utilized this system and first determined the functional profiles of *C*. *albicans*-specific CD4 T cells as compared to CMV-specific CD4 T cells in healthy human subjects. PBMC samples from healthy donors were labeled with CFSE, a fluorescent dye to track T cell division, and then stimulated with *C*. *albicans* or CMV antigen for 6 days, during which memory CD4 T cells underwent Ag-specific proliferation in response to stimulation. Cells were re-stimulated on day 6 for *de novo* cytokine synthesis. Functional profiles (IL-17, IL-22, IL-2, IFN-γ and MIP-1β) of *C*. *albicans-* or CMV-specific CD4 T cells in PBMCs were examined in CFSE-low CD4 T cells by multi-color flow cytometry (Fig A in S1 Appendix). Verification of the system has been described in previous reports [12, 34].

**Fig 1:**
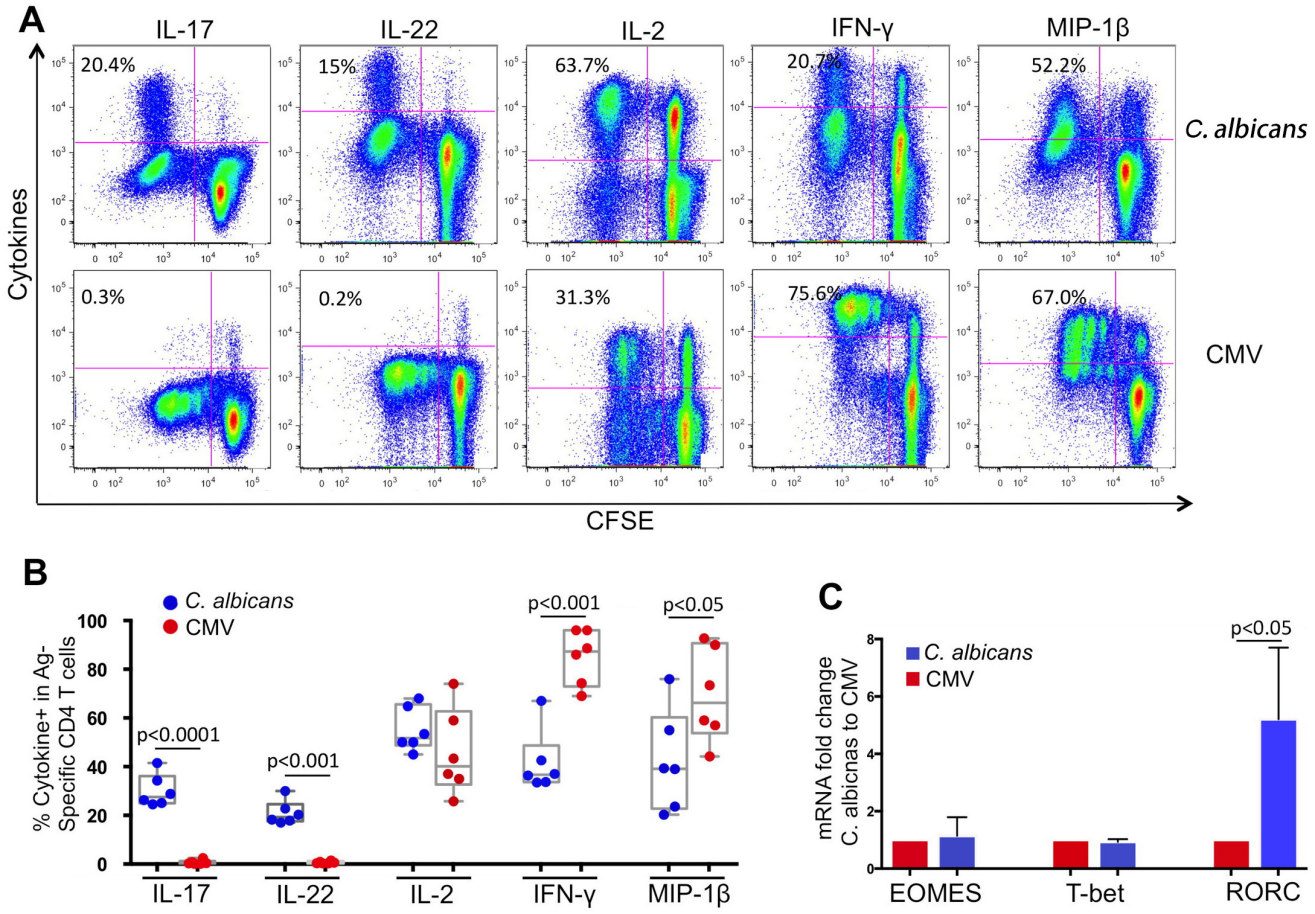
Functional characteristics of *C*. *albicans*- and CMV-specific CD4 T cells in healthy subjects. (A) Representative flow cytometric plots are shown for different cytokine expression in CFSE-low CD4+ T cells in PBMC after cognate antigen *C*. *albicans* (top) or CMV (bottom) stimulation. CFSE-labeled healthy donor PBMCs were stimulated with antigens for 6 days, followed by re-stimulation with PMA for *de novo* cytokine synthesis. CD3+CD4+ T lymphocytes are gated for analysis and the number in each plot indicates the percentage of CFSE-low Ag-specific CD4 T cells positive for each cytokine. (B) Comparison for percentages of Ag-specific CD4 T cells positive for each cytokine (cytokine+ CFSE-low%) between *C*. *albicans*- and CMV-specific CD4 T cells from multiple healthy subjects (n = 6). (C) Gene expression of Th1 (T-bet and EOMES) and Th17 (RORC) lineage-specific transcription factors between *C*. *albicans*- and CMV-specific CD4 T cells. Ag-specific CD4 T cells were sorted from PBMCs based on CFSE-low and subjected to RNA extraction and real-time PCR quantification. The data is shown as fold change for mRNA levels in *C*. *albicans*-specific CD4 T cells relative to CMV-specific CD4 T cells.

We found that *C*. *albicans*-specific CD4 T cells displayed a distinct functional profile from CMV-specific CD4 T cells in healthy donors (Fig 1A). Compared to CMV-specific CD4 T cells, which predominantly expressed Th1-associated cytokine IFN-γ (75.6%) and MIP-1β (67%), *C*. *albicans*-specific CD4 T cells expressed high levels of IL-17 (20.4%), IL-22 (15%) and IL-2 (63.7%), in addition to expression of IFN-γ and MIP-1β, suggesting a Th17/Th1-like phenotype for *C*. *albicans*-specific CD4 T cells in human subjects (Fig 1A). Analysis of PBMCs from multiple donors (n = 6) showed that expression of IL-17 (p<0.0001), IL-22 (p<0.001), IFN-γ (p<0.001) and MIP-1β (p<0.01) was statistically different between *C*. *albicans-* and CMV-specific CD4 T cells (Fig 1B). Poly-functional analysis showed that *C*. *albicans*-specific CD4 T cells demonstrate a more poly-functional profile and can co-express multiple cytokines compared to CMV-specific CD4 T cells (Fig B in S1 Appendix).

We also measured gene expression of Th17 and Th1 lineage-specific transcription factors, including RORC (Th17), T-bet and EOMES (Th1), in *C*. *albicans*- and CMV-specific CD4 T cells from the same donor PBMCs (Fig 1C). CFSE-low, CD4 T cells were sorted from PBMC and subjected to real-time PCR quantification. We found that while gene expression of Th1 transcription factors T-bet and EOMES was comparable between C. *albicans*- and CMV-specific CD4 T cells, the Th17 transcription factor RORC was expressed at significantly higher levels in *C*. *albicans*-specific CD4 T cells, further suggesting the mixed Th17/Th1-like phenotype of *C*. *albicans*-specific CD4 T cells in human subjects (Fig 1C).

Since no significant difference in T-bet and EOMES expression was observed at mRNA level between C. *albicans*- and CMV-specific CD4 T cells, we measured protein expression of these two transcription factors using flow cytometry and found that compared to CFSE-Hi non-specific CD4 T cells, both *C*. *albicans-* and CMV-specific CD4 T cells expressed higher levels of T-bet and EOMES, although the expression levels in CMV-specific CD4 T cells appeared to be slightly higher than those in *C*. *albicans*-specific CD4 T cells (Fig C in S1 Appendix). The results suggest that both Ag-specific CD4 T cell populations in this system manifest increased expression of Th1 transcription factors than non-specific CD4 T cells, which is in line with previous reports showing that T-bet and EOMES were readily detectable in CMV-specific CD4 T cells albeit at lower level than in their CD8 counterparts [35–37].

### Th17-like functional subsets of *C*. *albicans*-specific CD4 T cells expressing IL-17, IL-22 and IL-2 are more susceptible to HIV infection *in vitro* than Th1-like subsets expressing IFN-γ and MIP-1β

**Fig 2:**
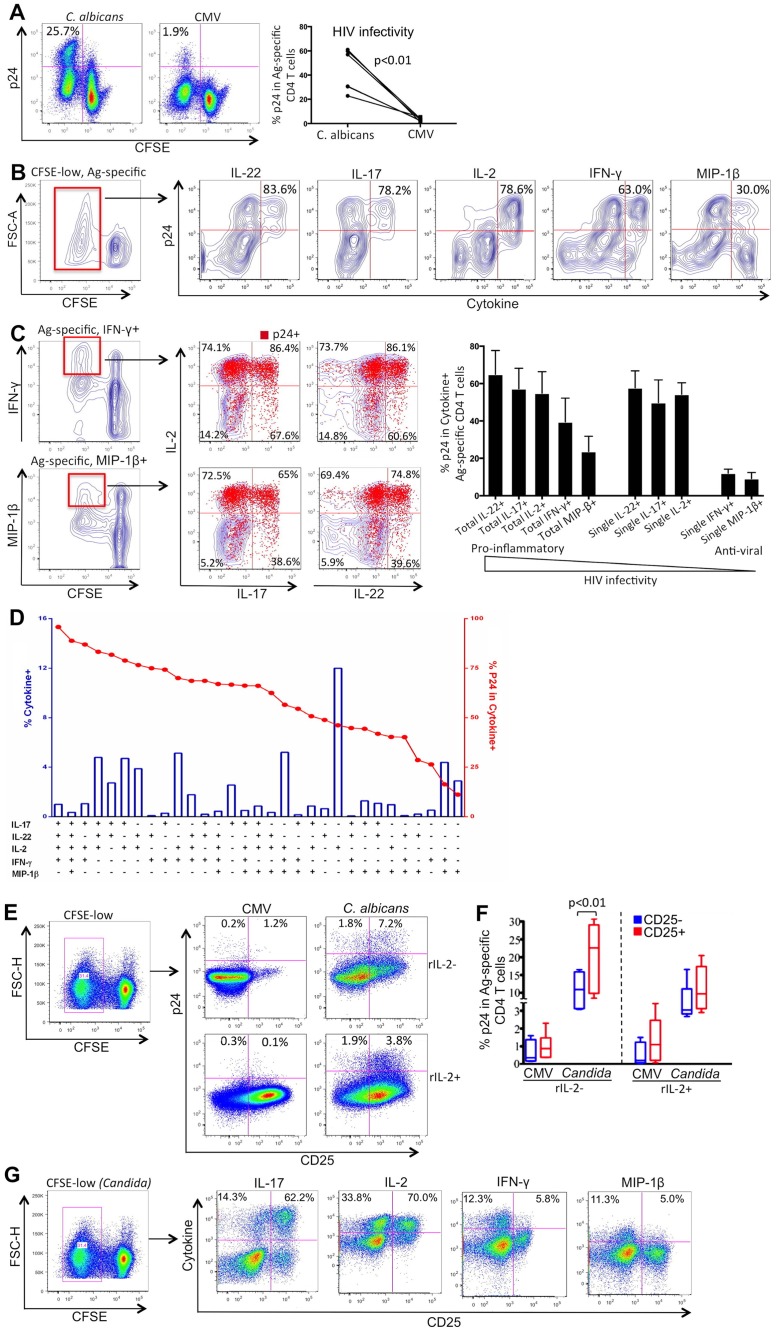
HIV infectivity in functional subsets of *C*. *albicans*-specific CD4 T cells. (A) Overall in vitro HIV susceptibility of *C*. *albicans*- and CMV-specific CD4 T cells in the same healthy donor PBMCs. CFSE-labeled PBMCs were stimulated with *C*. *albicans* (left) or CMV (right) for 3 days, and exposed to HIV for infection. HIV infection of Ag-specific CD4 T cells was examined as intracellular p24+ rates in CFSE-low CD4 T cells (number in top-left quadrant). Representative flow cytometric data (left) and cumulative results (right) are shown. (B) Flow cytometric analysis of HIV infection in each cytokine+ subset of *C*. *albicans*-specific CD4 T cells. CFSE-low, *C*. *albicans*-specific CD4 T cells were gated (left) and co-expression of intracellular p24 and cytokine within the gated population was analyzed (right). Number in top-right quadrant shows p24+ percentage in each cytokine+ population. (C) Analysis of HIV infectivity (p24+, red dot) in IFN-γ- or MIP-1β-producing, *C*. *albicans*-specific CD4 T cells with or without co-expression with IL-2, IL-17 or IL-22 (blue background). Number in each quadrant shows p24+% in each functional subset of *C*. *albicans*-specific CD4 T cells and calculated using FlowJo program. Comparison of HIV infectivity (p24+%) in different functional subsets of *C*. *albicans*-specific CD4 T cells is shown (n = 6). (D) Boolean gating and spice analysis for HIV infectivity (p24+%) in all different functional subsets of *C*. *albicans*-specific CD4 T cells. (E) Analysis of CD25 expression and HIV infectivity in *C*. *albicans* and CMV-specific CD4 T cells with (top) and without (bottom) exogenous rIL-2. Number in each quadrant shows p24+% in CD25- or CD25+ subset of CFSE-low, Ag-specific CD4 T-cell population. (F) Cumulative data comparing p24+% in CD25- and CD25+ subset for multiple subjects (n = 6). (G) Analysis of CD25 and cytokine co-expression in *C*. *albicans*-specific CD4 T cells. Two-tailed p values are denoted.

Based on this system, we examined HIV susceptibility of *C*. *albicans*- and CMV-specific CD4 T cells from healthy donor PBMCs and found that *C*. *albicans*-specific CD4 T cells were substantially more permissive to HIV than CMV-specific CD4 T cells *in vitro* (Fig 2A), a finding that was consistent with our previous report [12]. To explore whether the significant difference in HIV susceptibility between *C*. *albicans*- and CMV-specific CD4 T cells is due to higher permissiveness of *C*. *albicans*-specific CD4 T cells or enhanced protection of CMV-specific CD4 T cells, we compared their HIV susceptibility with that of non-specific total CD4 T cells that were globally stimulated with anti-CD3/CD28 (Fig D in S1 Appendix), and found that HIV infectivity in globally stimulated CD4 T cells fell into the range between *C*. *albicans*- and CMV-specific CD4 T cells (Fig D in S1 Appendix), implying that the difference in HIV susceptibility between *C*. *albicans*- and CMV-specific CD4 T cells might attribute to the combination of both. This will be further investigated subsequently.

Functional characteristics of CD4 T cells have been shown to associate with their susceptibility to HIV [10, 13]. In order to define the relationship between HIV infectivity and functional characteristics for *C*. *albicans*-specific CD4 T cells, we performed the HIV susceptibility assay as described in Fig A in S1 Appendix. Healthy donor PBMCs were CFSE-labeled and stimulated with *C*. *albicans* antigen, followed by exposure to HIV. Three days after HIV infection, cells were re-stimulated with PMA/ionomycin and subjected to comprehensive flow cytometric analysis (Fig A in S1 Appendix). Cytokine expression in activated T cells is transient and the CFSE-low, Ag-specific CD4 T cells in this system undergo days of proliferation. In order to simultaneously measure functional characteristics (cytokine production) and HIV infectivity (intracellular p24), cells were re-stimulated with the global PMA/ionomycin stimulus on day 6 for cytokine re-synthesis in T cells.

As shown in Fig 2B, by gating on CFSE-low CD4 T-cell population, we determined HIV infectivity in each functional subset of *C*. *albicans*-specific CD4 T cells by measuring co-expression of intracellular HIV p24, as an indication of productive HIV infection, with individual cytokines (Fig 2B). Number in each plot showed intracellular p24+ rate in cytokine-producing, *C*. *albicans*-specific CD4 subset. We found that the IL-22, IL-17 or IL-2 functional subsets of *C*. *albicans*-specific CD4 T cells were more susceptible to HIV as compared to those subsets expressing MIP-1β and IFN-γ (Fig 2B). We measured expression of CCR5, an important co-receptor for HIV entry, on these different functional CD4 subsets and no significant difference was observed (Fig E in S1 Appendix). Instead, we found that the higher HIV infectivity in IL-22+, IL-17+ and IL-2+ subsets appeared to be associated with their lower levels of MIP-1β co-expression (Fig F in S1 Appendix). This observation was consistent with an earlier report showing that *in vitro* differentiated IL-17+ CD4 T cells are more susceptible to HIV than IFN-γ+ CD4 T cells due to reduced expression of MIP-1β [38].

We noted that HIV infectivity in IFN-γ+ and MIP-1β+ subsets was also fairly high (63% and 30%, respectively) (Fig 2B). Next, we gated on the IFN-γ+ or MIP-1β+, CFSE-low CD4 subsets (Fig 2C) and found that significant fractions of IFN-γ+ (or MIP-1β+) subset co-express with IL-2 and IL-17 or IL-22 (Fig 2C and Fig G in S1 Appendix). When we further performed intracellular p24 (red dots) and cytokine (blue background) overlaying analysis, as shown in Fig 2C, we identified that HIV predominantly infected IFN-γ+ or MIP-1β+ CD4 subsets that co-expressed IL-2 and IL-17 or IL-22; the single IFN-γ- or MIP-1β-producing CD4 subsets (bottom left quadrant) demonstrated very low HIV infectivity (Fig 2C). In order to better differentiate HIV infectivity in all different functional subsets (combination of cytokine+), we performed comprehensive Boolean gating and spice analysis. As shown in Fig 2D, we identified a consistent trend that HIV infectivity (p24+%) was substantially higher in populations that express of IL-17, IL-2 and IL-22, but lower in populations that only express MIP-1β and/or IFN-γ.

CD25, the high-affinity chain of IL-2 receptor, was shown to be important for HIV infection of CD4 T cells *in vitro* [13, 39]. We examined expression of CD25 on *C*. *albicans-* and CMV-specific CD4 T cells and evaluated its relationship with HIV infectivity and cytokine expression in our system (Fig 2E–2G). Interestingly, the data showed that *C*. *albicans-*specific CD4 T cells expressed substantially higher level of CD25 than CMV-specific CD4 T cells (Fig 2E). Importantly, productive HIV infection was predominantly observed in CD25+ subset both *C*. *albicans-* and CMV-specific CD4 T cells (Fig 2E, top panels; Fig 2F). We also examined the impact of exogenous IL-2 on CD25 expression and HIV infectivity (Fig 2E, bottom panels). Despite recombinant IL-2 (rIL-2) induced significant increase in CD25 expression and CD4 T-cell proliferation, HIV infectivity in exogenous IL-2-treated cells were not enhanced (Fig 2E, bottom; Fig 2F), indicating that HIV infectivity is associated with endogenous IL-2-CD25 signaling. In addition, we investigated relationship between CD25 and cytokine expression in *C*. *albicans*-specific CD4 T cells. As shown in Fig 2G, CD25 predominantly co-expressed with IL-17, IL-22 and IL-2, but not IFN-γ- or MIP-1β, which is consistent with the observation about HIV infectivity in different CD4 T-cell subsets. Taken together, these data suggest that compared to CMV, *C*. *albicans*-specific CD4 T cells manifest distinct phenotypic and functional characteristics that favor productive HIV infection in these cells.

### Earlier impairment of IL-17, IL-22 and IL-2 functional response of *C*. *albicans*-specific CD4 T-cells in ART naïve, HIV-infected individuals

As described earlier, CD4-mediated cellular immunity is a predominant host defense mechanism for immune control of pathogenic *C*. *albicans* infection [23–27]. While Th1 response was initially thought to be the key mediator of immunity, more recent studies have supported critical role of IL-17- and IL-22-producing Th17, but not IFN-γ-producing Th1, response in protection against candidiasis [31, 32] [33]. After showing that IL-17, IL-22 and IL-2 functional subsets of *C*. *albicans*-specific CD4 T cells were more permissive to HIV *in vitro*, we next aimed to determine the *in vivo* impact of HIV on *C*. *albicans*-specific CD4 T-cell immunity and the associated functional subsets in HIV-infected individuals. To do so, we selected HIV-infected subjects in RV21 cohort who manifested ongoing CD4 depletion, which permitted us to longitudinally examine the *in vivo* effect of HIV on pathogen-specific CD4 cells. We identified 20 HIV-infected subjects with positive responses to both *C*. *albicans* and CMV antigens and the PBMC samples from these subjects were accordingly investigated (Table 1). To better explore the impact of HIV on pathogen-specific CD4 T-cell immunity, multiple assays were performed. Due to limited cell number for each HIV-infected subject, we appropriately allocated cell samples from these 20 subjects for different assays as detailed below.

**Table 1:** Characteristics of HIV-infected subjects from the RV21 cohort*.

Subject	Stages	ART Treatment	PBMC	CD4%	CD4 count
1	Early	No	Yes	40	1007
	Chronic	No	Yes	30	561
2	Early	No	Yes	46	1103
	Chronic	No	Yes	45	444
3	Early	No	Yes	34	1233
	Chronic	No	Yes	25	543
4	Early	No	Yes	33	783
	Chronic	No	Yes	11	130
5	Early	No	Yes	28	776
	Chronic	No	Yes	18	257
6	Early	No	Yes	40	794
	Chronic	No	Yes	18	260
7	Early	No	Yes	25	585
	Chronic	No	Yes	15	290
8	Early	No	Yes	36	576
	Chronic	No	Yes	12	86
9	Early	No	Yes	27	624
	Chronic	No	Yes	27	346
10	Early	No	Yes	29	523
	Chronic	No	Yes	15	160
11	Early	No	Yes	29	716
	Chronic	No	Yes	21	241
12	Early	No	Yes	34	560
	Chronic	No	Yes	20	215
13	Early	No	Yes	26	456
	Chronic	No	Yes	15	189
14	Early	No	Yes	37	819
15	Early	No	Yes	55	621
16	Early	No	Yes	25	631
	Chronic	No	Yes	7	75
17	Early	No	Yes	51	1146
	Chronic	No	Yes	31	429
18	Early	No	Yes	33	807
	Chronic	No	Yes	21	241
19	Early	No	Yes	36	927
	Chronic	No	Yes	24	361
20	Early	No	Yes	32	530
	Chronic	No	Yes	15	114
**Mean CD4 counts**				**Early**	**797**
				**Chronic**	**251**

* HIV-infected subjects with progressive CD4 T cell depletion and positive responses to measured antigens were selected for the study

**Fig 3:**
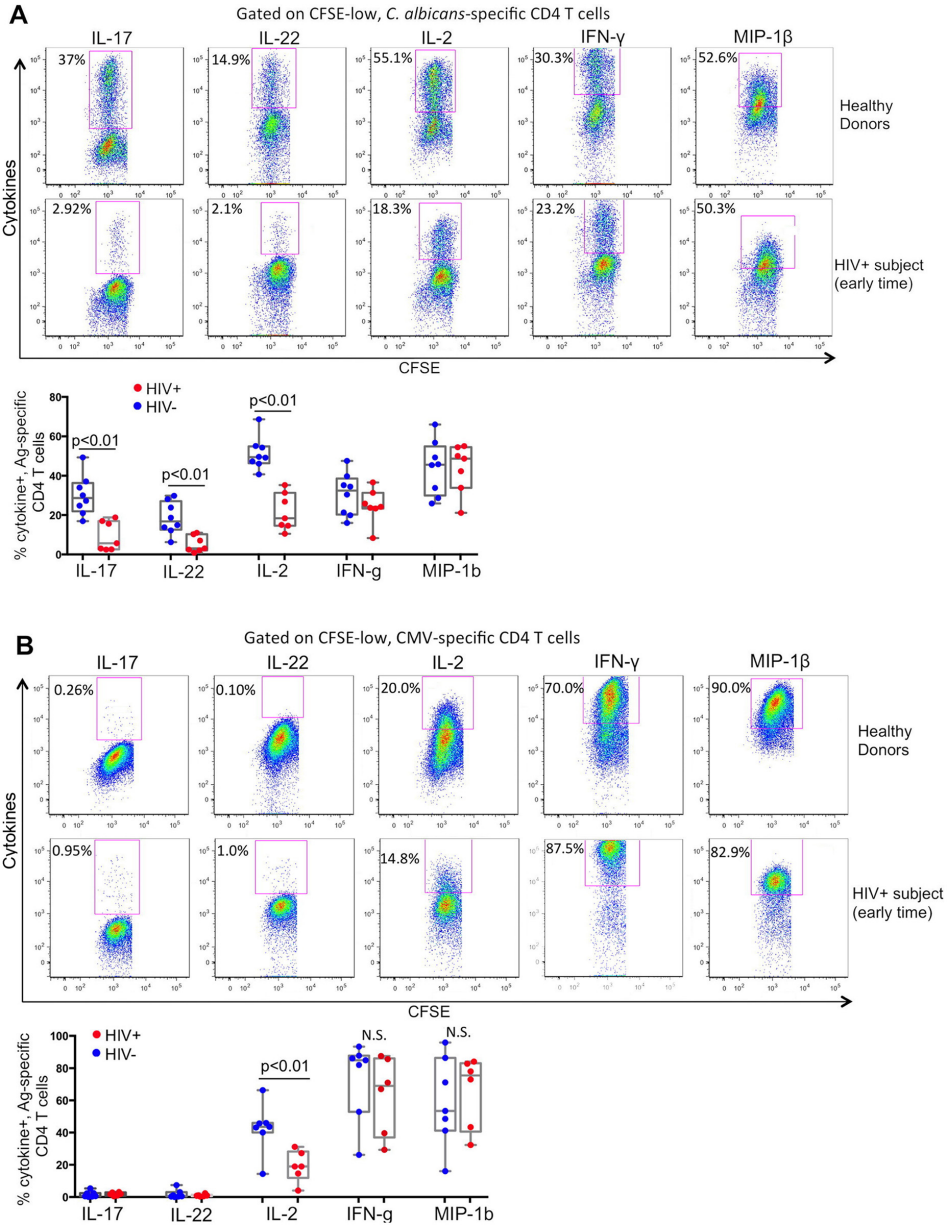
Functional profiles of *C*. *albicans*- and CMV-specific CD4 T-cell responses in healthy donors and HIV-infected subjects. (A) Functional profile of *C*. *albicans*-CD4 T cell responses. Representative flow cytometry data showing expression of each cytokine in CFSE-low, *C*. *albicans*-specific CD4 T cells in PBMCs of un-infected healthy (top) or HIV-infected (bottom) subjects. PBMCs of HIV-infected subjects used here were collected during early infection before massive CD4 depletion occurred. Shown are the gated CFSE-low CD4 T cells and the number in each plot shows cytokine+ % in CFSE-low *C*. *albicans*-specific CD4 T cells. Comparison for percentage of *C*. *albicans*-specific CD4 T cells positive for each cytokine from multiple un-infected and HIV-infected subjects (n = 7) is shown. (B) Functional profile of CMV-specific CD4 T cell responses. Representative flow cytometry data showing expression of each cytokine in CFSE-low, CMV-specific CD4 T cells in PBMCs of un-infected healthy (top) or HIV-infected (bottom) subjects. Comparison for multiple un-infected (n = 7) and infected subjects (n = 6) is also shown. Two-tailed p values are denoted. N.S. represents non-significant.

We first used a similar method as described in Fig 1 and measured functional profiles of proliferating *C*. *albicans*-specific CD4 T cells in the HIV-infected subjects as compared to healthy donors (Fig 3). PBMCs of HIV+ subjects measured here were collected at early HIV infection when profound CD4 depletion had not occurred and *C*. *albicans*-specific response remained detectable. As shown in Fig 3A, only the CFSE-low, *C*. *albicans*-specific CD4 T cell populations were gated for analysis. Interestingly, we found that compared to *C*. *albicans*-specific CD4 T cells in control PBMC of healthy donors, which manifested strong proliferative response and normal production of all cytokines tested (IL-17, IL-22, IL-2, IFN-γ and MIP-1β), *C*. *albicans*-specific CD4 T cells from HIV-infected subjects, despite being able to proliferate at comparable levels, demonstrated a preferential impairment in Th17 functions with substantial decrease in IL-17, IL-22 and IL-2 production, while their Th1 response (expression of IFN-γ or MIP-1β) was not significantly affected (Fig 3A). We examined PBMC samples from multiple subjects (n = 7) and observed statistically significant differences for expression of IL-17 (p<0.01), IL-22 (p<0.01) and IL-2 (p<0.01), but not IFN-γ (N.S.) or MIP-1β (N.S.) between healthy donors and HIV-infected subjects (Fig 3A). Importantly, we also measured functional profile of CMV-specific CD4 T cells in these HIV-infected subjects (Fig 3B) and found that CMV-specific CD4 T cells at early HIV infection manifest a comparable functional profile with that in uninfected healthy donors; no significant reduction in expression of Th1 cytokines IFN-γ (N.S.) and MIP-1β (N.S.) was observed, although the IL-2 expression in CMV-specific CD4 T cells appeared to be impaired in HIV-infected subjects as compared to healthy subjects (p<0.01) (Fig 3B). Taken together, these data from HIV-infected subjects were consistent with the *in vitro* observations in Fig 2 and imply that there was a sequential dysfunction for *C*. *albicans*-specific CD4 T-cell response with earlier impairment of Th17 functions during HIV infection. Given the critical role of Th17, but not Th1, response in protection for mucosal candidiasis, this finding is potentially significant and suggests that early following HIV infection, the anti-*C*. *albicans* specific immunity might become rapidly ineffective, despite their proliferative and Th1-type responses are still readily detectable.

### *C*. *albicans*-specific CD4 T cells harbor modestly but significantly higher levels of HIV DNA than CMV-specific CD4 T cells in HIV-infected subjects

**Fig 4:**
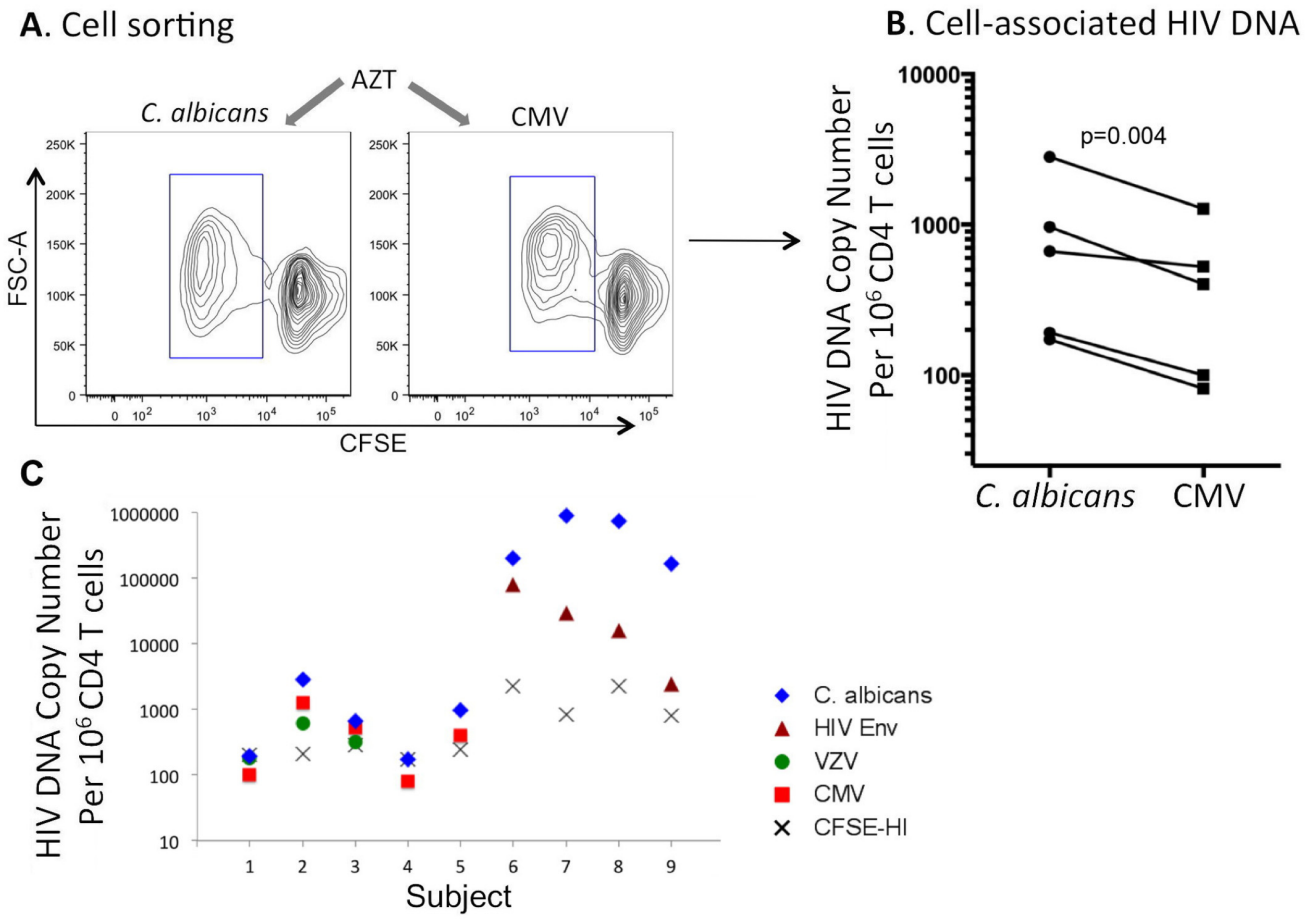
Quantification of cell-associated HIV DNA in sorted Ag-specific CD4 T cells of HIV-infected subjects. (A) PBMCs of untreated HIV-infected subjects (early during infection before massive CD4 depletion occurred) were CFSE-labeled and stimulated with *C*. *albicans* or CMV antigen in the presence of AZT, to prevent *de novo* HIV replication, for 5 days. *C*. *albicans*- and CMV-specific CD4 T cells were sorted from PBMCs based on CFSE-low. (B) Cell-associated HIV DNA in sorted CD4 T cells was quantified by real-time PCR and compared between *C*. *albicans* and CMV antigens (n = 5). (C) Comparison of cell-associated HIV DNA in *C*. *albicans*-specific CD4 T cells with that in CMV-specific (red), VZV-specific (green), HIV Env-specific (purple) and CFSE-Hi non-specific (gray) CD4 T cells within the same HIV-infected individuals.

We have shown in Fig 2A that *C*. *albicans*-specific CD4 T cells are more susceptible to HIV infection than CMV-specific CD4 T cells *in vitro* [12]. In order to determine if this occurs *in vivo*, we examined the levels of cell-associated HIV DNA using quantitative PCR in *C*. *albicans*- and CMV-specific CD4 T cells from HIV-infected subjects [13, 40]. To do so, we used PBMC samples of HIV-infected subjects collected at early HIV infection when profound CD4 depletion had not occurred and both *C*. *albicans*- and CMV-specific CD4 T cell responses were detectable. PBMCs were CFSE-labeled and stimulated with *C*. *albicans*- or CMV-antigen for 5 days, during which HIV replication inhibitor AZT was added to cell culture to prevent potential *de novo* HIV replication. No HIV virus was detected in the culture supernatants after antigen stimulation and T-cell division, supporting the effectiveness of AZT in blocking possible *de novo* viral replication (Fig H in S1 Appendix). After stimulation, *C*. *albicans*- and CMV-specific CD4 T-cell populations from the same PBMCs were sorted based on CFSE-low (Fig 4A) and then subjected to quantification of HIV DNA. Plasmids encoding HIV Gag or GAPDH were used to generate standard curves for quantifying real copy numbers of HIV DNA in the sorted Ag-specific CD4 T cells (Fig I in S1 Appendix). Quantification of HIV DNA in the sorted cells was normalized to GAPDH and expressed as copy number/10^6^ CD4 T cells.

As shown in Fig 4B, *C*. *albicans-*specific CD4 T cells harbored modestly but significantly higher levels of HIV DNA than CMV-specific CD4 T cells (p = 0.004). We measured PBMCs from 5 HIV-infected individuals and observed a consistent trend, although the difference for some subjects was modest (Fig 4B). Since predominant sites of HIV infection and replication are lymphoid or mucosal tissues, not peripheral blood, we speculate that differences for HIV DNA content between *C*. *albicans-* and CMV-specific CD4 T cells, when isolated from these effector sites, might be more profound.

Quantification of cell-associated HIV DNA to evaluate *in vivo* HIV susceptibility has been previously reported for MTB- and HIV-specific CD4 T cells [13, 40]. To gain better insights into relative HIV susceptibility of different Ag-specific CD4 T cells *in vivo* in infected individuals in the absence of viral suppression, we further measured additional antigens for comparison with *C*. *albicans*. Since the HIV-infected subjects examined in this study resided in the US and demonstrated very low MTB response, we were unable to directly compare *C*. *albicans* with MTB. Instead, we measured CD4 T cells specific for varicella zoster virus (VZV), another herpes virus similar to CMV, as well as CD4 T cells specific for HIV Env protein; HIV DNA in CFSE-Hi non-specific CD4 T cells from the same individuals was also compared (Fig 4C). Since cell number for each subject was limited and some subjects only responded to certain antigens, not all antigens were compared for each subject. Interestingly, we found that among the subjects investigated, *C*. *albicans*-specific CD4 T cells appeared to also harbor higher levels of HIV DNA as compared to VZV-specific (subject 1–3) and HIV Env-specific (subject 6–9) CD4 T cells (Fig 4C). Of note, HIV DNA copies in *C*. *albicans*-specific CD4 T cells varied fairly substantially among different subjects, an observation that was also reported for MTB-specific CD4 T cells [13]. Taken together, these results suggest that *C*. *albicans*-specific CD4 T cells are highly susceptible to HIV *in vivo* when compared to multiple other antigens.

### *C*. *albicans*-specific CD4 T cells display strong mucosal homing phenotype

**Fig 5:**
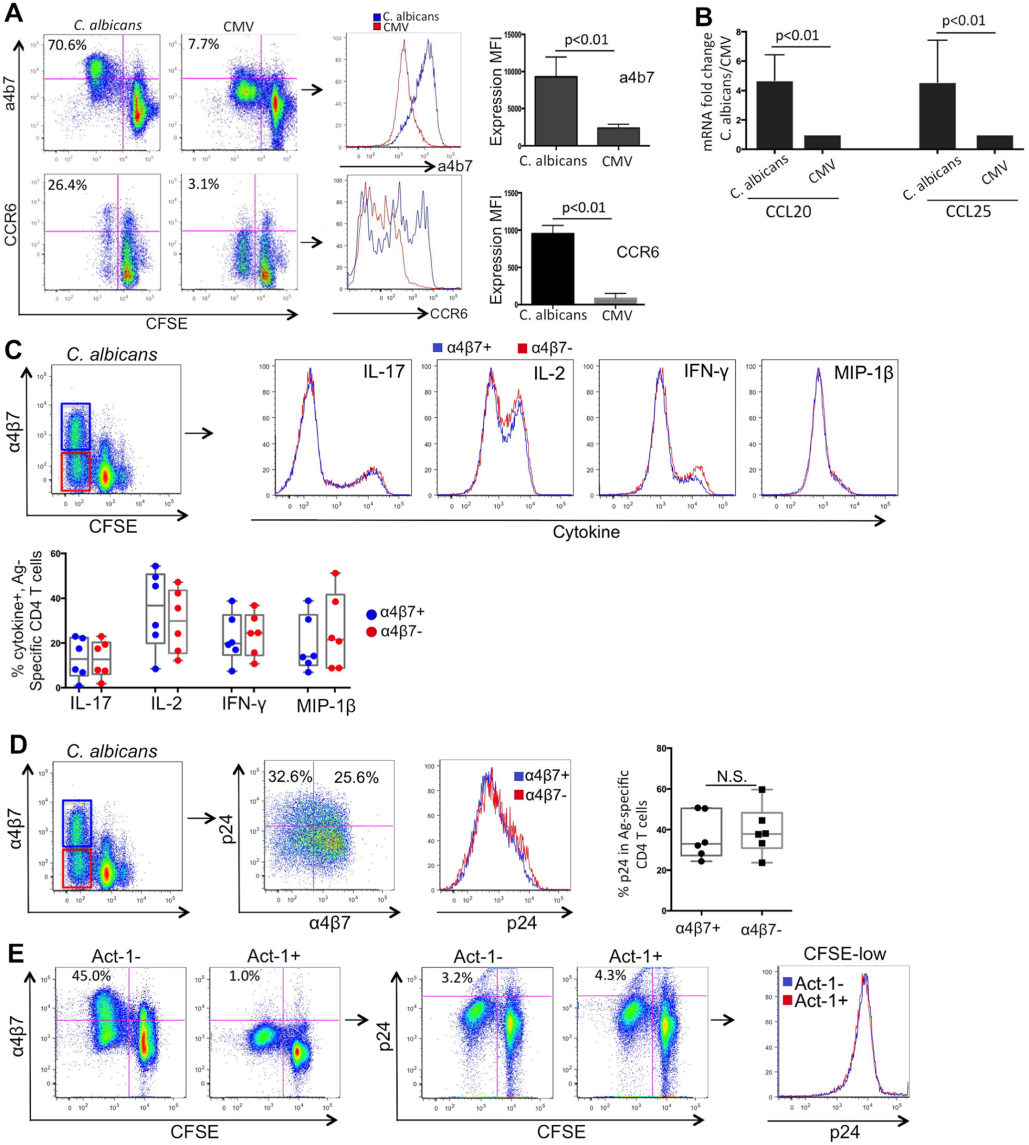
Analysis of mucosal homing markers of *C*. *albicans*-specific and CMV-specific CD4 T cells. (A) Representative flow cytometry dot plots (left) and histogram comparison (middle) for expression of α4β7 and CCR6 between *C*. *albicans*- and CMV-specific CD4 T cells are shown. Cumulative results for comparing mean fluorescence intensity (MFI) of α4β7 and CCR6 expression between *C*. *albicans*- and CMV-specific CD4 T cells is also shown (right) (n = 6). (B) Gene expression of mucosal homing chemokines CCL-20 and CCL-25 in sorted *C*. *albicans*- and CMV-specific CD4 T cells. The data is shown as fold change for *C*. *albicans*-specific CD4 T cells relative to CMV-specific CD4 T cells. (C) Comparison of cytokine expression in α4β7+ (blue) and α4β7- subsets (red) of *C*. *albicans*-specific CD4 T cells. Representative histogram (top) and cumulative results for comparing cytokine+% between α4β7+ and α4β7- subset (bottom) are shown. (D) Comparison of HIV infectivity (p24+%) in α4β7+ and α4β7- subsets of *C*. *albicans*-specific CD4 T cells. Both representative and cumulative results are shown. (E) Impact of α4β7 blocking by ACT-1 antibody on HIV infectivity in *C*. *albicans*-specific CD4 T cells.

As discussed above, compared to peripheral blood, mucosal tissues represent a preferential site for HIV infection and manifest most remarkable CD4 depletion at all stages of HIV disease [41, 42]. Integrin α4β7 is an important mucosal homing receptor, directing migration of CD4 T cells from blood to gut, and CCR6 is a marker associated with Th17 cells that contributes to their migration to mucosal tissues [43]. We examined expression of these mucosal homing receptors on *C*. *albicans*- and CMV-specific CD4 T cells from the HIV-infected subjects. Our data showed that significant fraction of *C*. *albicans*-specific CD4 T cells expressed high levels of α4β7 and CCR6, while CMV-specific CD4 T cells rarely expressed these two receptors (*C*. *albicans* vs. CMV: 70.6% to 7.7% for α4β7; 26.4% vs. 3.1% for CCR6) (Fig 5A).

We also examined gene expression of CCL-20 and CCL-25, two important mucosal homing chemokines [43, 44], in *C*. *albicans-* and CMV-specific CD4 T cells. As described earlier (Fig A in S1 Appendix), Ag-specific CD4 T cells were sorted from PBMCs based on CFSE-low and expression of these two genes was quantified by real-time PCR. We found that consistent with expression of mucosal homing receptors, *C*. *albicans-*specific CD4 T cells also expressed significantly higher levels of CCL-20 and CCL-25 than CMV-specific CD4 T cells (Fig 5B). These data altogether indicate that in accordance with their Th17-like phenotype, *C*. *albicans*-specific CD4 T cells demonstrate a strong mucosal homing potential and may be more likely to migrate to mucosal tissues in HIV-infected subjects.

α4β7 integrin can directly interact with HIV surface protein gp120 [45]. Although the role of α4β7 in HIV pathogenesis is not fully clear, it has been suggested that strong α4β7 reactivity may provide an increased fitness for mucosal HIV transmission [45, 46]. We next investigated potential impact of α4β7 on cytokine expression and HIV infectivity in *C*. *albicans*-specific CD4 T cells. We found that IL-17, IL-2, IFN-γ and MIP-1β were expressed at comparable levels between α4β7+ and α4β7- subsets (Fig 5C). Analysis of α4β7 expression and HIV infectivity showed no significant difference in HIV infection between α4β7+ and α4β7- subsets as well (Fig 5D). In addition, we used ACT-1, the anti-human α4β7 antibody known to efficiently block binding of HIV gp120 to α4β7 [47], to block the interaction between HIV and α4β7 during HIV infection. The data showed that pre-inculcation of PBMCs with ACT-1 led to reduced staining of *C*. *albicans*-specific CD4 T cells for α4β7 (Fig 5E); however, blocking α4β7 could not reduce HIV infection of *C*. *albicans*-specific CD4 T cells (Fig 5E). Taken together, our results suggest that unlike CD4 and CCR5, α4β7 may not be required for *in vitro* HIV replication in our system, which is consistent with some previous reports [48].

### Preferential and progressive loss of *C*. *albicans*-specific CD4 T-cell response in ART naïve, HIV-infected subjects with ongoing CD4 depletion

**Fig 6:**
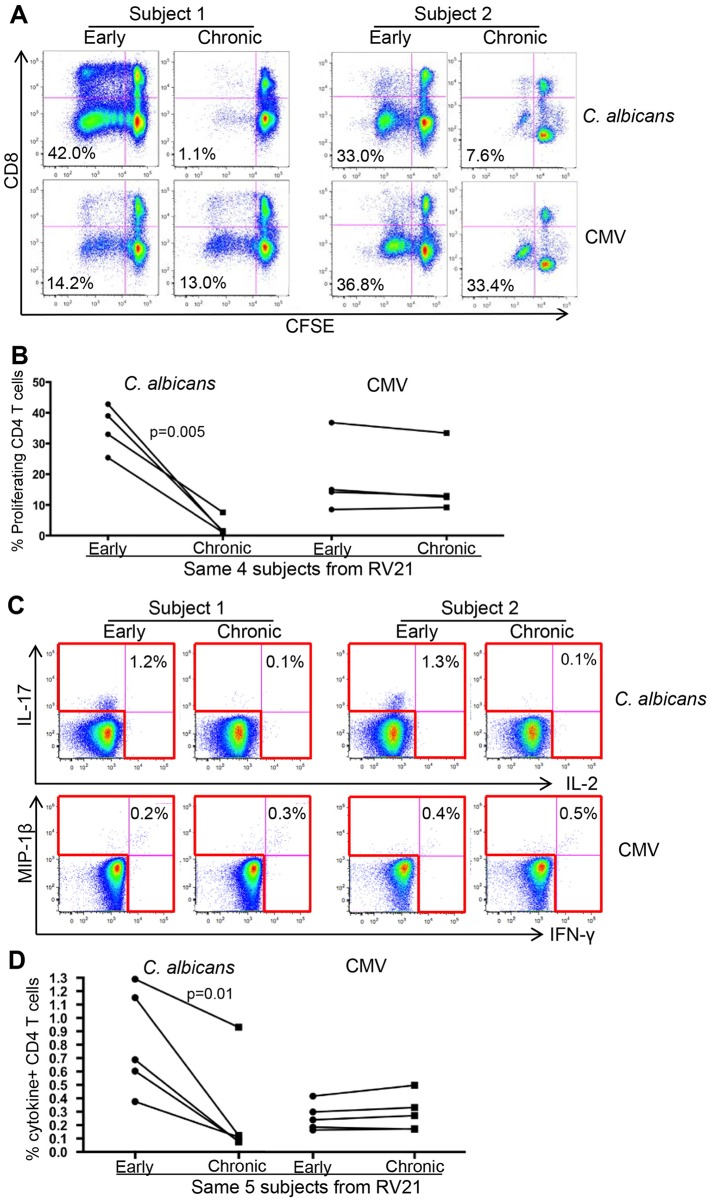
Preferential and progressive depletion of *C*. *albicans*-specific CD4 T-cell response in HIV-infected subjects. (A) Representative flow cytometry data (2 subjects) showing *C*. *albicans*- (top) and CMV-specific (bottom) CD4 T-cell proliferative responses within the same PBMCs from HIV-infected subjects who manifested ongoing CD4 depletion. PBMCs collected at early infection (high CD4 count) and chronic infection (low CD4 count) from the same subjects are shown (gated on CD3+ T cells). (B) Longitudinal magnitudes of *C*. *albicans*- (left) and CMV- (right) CD4 T-cell proliferative responses from multiple HIV-infected subjects (n = 4) are shown. (C) Short-term *ex vivo* stimulation of PBMCs with peptide pools (*C*. *albicans* MP65 and CMV pp65) and intracellular cytokine staining for measuring frequencies of Ag-specific CD4 T cells in PBMCs. Representative data from 2 subjects show cytokine expression in *C*. *albicans*- (IL-17 and IL-2) and CMV-specific (IFN-γ and MIP-1β) CD4 T cells. The number in each plot shows total percentage of CD4 T cells expressing one and both cytokines. (D) Longitudinal frequencies of *C*. *albicans*- (left) and CMV- (right) CD4 T cells from multiple HIV-infected subjects (n = 5) are shown. Two-tailed p values are denoted.

In order to investigate longitudinal impact of HIV on *C*. *albicans-* and CMV-specific CD4 T-cell immunity *in vivo*, we first measured the proliferative responses of *C*. *albicans-* and CMV-specific CD4 T cells in PBMCs that were collected at early (mean CD4 count: 797) and chronic (mean CD4 count: 251) stages of HIV infection with time intervals of 2–6 years from the same HIV-infected individuals (Table 1). Ag-specific CD4 T-cell proliferative response in PBMCs was measured using the similar method as described in Fig 1. Since PBMCs were stimulated with whole *C*. *albicans* or CMV antigens, predominantly CD4, but not CD8, T-cell proliferative response was induced (Fig 6A). Since *in vitro* antigen stimulation can lead to significant down-regulation of CD4 receptor, we used CD3+CD8- phenotype to identify CD4 T-cell population following antigen stimulation (Fig 6A). Interestingly, among the subjects examined, we found that while the CMV-specific CD4 T-cell proliferative response was persistent and comparable between early and chronic stages (early vs. chronic: 14.2% to 13%; subject 1), the *C*. *albicans*-specific CD4 T-cell proliferative response from the same subjects, which was readily detectable at high magnitudes at early HIV infection, was preferentially lost at late HIV infection (early vs. chronic: 42% to 1.1%; subject 1) (Fig 6A). Significant difference for magnitudes of *C*. *albicans*-specific CD4 T-cell proliferative responses between early and chronic stages was observed (n = 4) (p = 0.005) (Fig 6B).

In order to compare the *de novo* frequencies of *C*. *albicans-* and CMV-specific CD4 T cells in PBMCs of HIV-infected subjects, we performed short-term antigen stimulation (overnight), where PBMCs were stimulated with peptide pools derived from *C*. *albicans* (MP65) or CMV (pp65), followed by intracellular cytokine staining. Expression of cytokines (IL-17 and IL-2 for *C*. *albicans*; IFN-γ and MIP-1β for CMV) was used to determine the frequencies of Ag-specific CD4 T cells in PBMCs. As shown in Fig 6C, while the *C*. *albicans*-specific CD4 T cells were readily detectable at fairly high levels early after HIV infection, they were lost or greatly reduced at late stage of HIV infection (p = 0.01); in contrast, the CMV-specific CD4 T cells were well maintained at comparable levels in both early and late stage of HIV infection from the same HIV-infected individuals (Fig 6C and 6D). The results are very consistent with the proliferation data and altogether provide strong evidence for preferential and progressive depletion of *C*. *albicans*-specific CD4 T-cell response in progressive HIV-infected subjects.

## Discussion

A better understanding of how pathogen-specific CD4 T cells are infected and/or depleted during HIV infection can provide important clinical insights into host susceptibility to opportunistic infections in AIDS patients. In this study, we used PBMC samples from an ART naïve, longitudinal HIV-infection cohort and reported a sequential dysfunction and preferential depletion of *C*. *albicans*-specific CD4 T-cell response, as compared to CMV-specific CD4 T-cell response, in HIV-infected individuals. Our results showed that *C*. *albicans-*specific CD4 T cells harbored higher levels of HIV DNA, which supports our *in vitro* findings and provides *in vivo* evidence for higher susceptibility of *C*. *albicans-*specific CD4 T cells to HIV. Such difference in HIV susceptibility may significantly contribute to their differential depletion rates *in vivo*. Also importantly, we identified an earlier impairment of Th17-associated functions (IL-22, IL-17 and IL-2) of *C*. *albicans*-specific CD4 T cells at early HIV infection when their proliferative and Th1 responses remain detectable, suggesting that anti-*C*. *albicans* cellular immunity may rapidly become inefficient early following HIV infection.

*C*. *albicans* commensalism in healthy individuals stimulates robust cellular immune responses. Many studies have shown that *C*. *albicans*-specific CD4 T-cell response serves as the predominant host defense mechanism for protection [20, 26, 49]. However, specific functional facets of anti-*C*. *albicans* CD4 T-cell responses responsible for immune control have been obscure. Earlier studies reported that Th1 response was the key mediator of immunity, based on the observation that deficiency of IL-12 p40 subunit in mice was associated with susceptibility to *C*. *albicans*; however, studies also showed that mice deficient in IFN-γ were still resistant to candidiasis [31]. It was later recognized that IL-12 shares the p40 subunit with IL-23, which promotes the differentiation of Th17 subset of CD4 T cells [32], suggesting a role for Th17, but not Th1, response in protection against candidiasis [25]. In support, a recent study by Santos *et al*. showed that Th17 CD4 cells confer the long-term adaptive immunity to oral *C*. *albicans* infections in a murine model [33]. Th17 cells produce two major cytokines, IL-17 and IL-22, which function to mobilize neutrophils and to enhance mucosal epithelial integrity respectively, and are shown to play important roles in host defense against mucosal candidiasis [50–53]. In our study, we identified that in the setting of HIV infection, *C*. *albicans-*specific CD4 T-cell responses manifest a sequential dysfunction with Th17-like functions (IL-17, IL-22 and IL-2) being impaired earlier following HIV infection as compared to Th1 functions (IFN-γ and MIP-1β) (Fig 3). In support, *in vitro* HIV susceptibility analysis showed that the IL-17, IL-22 and IL-2 functional subsets of *C*. *albicans-*specific CD4 T cells are more permissive to HIV than the IFN-γ and MIP-1β subsets (Fig 2). These data suggest that during HIV infection anti-*C*. *albicans* CD4 T-cell immunity, even in the presence of detectable proliferative and Th1 responses, might quickly become less efficient due to preferential impairment of Th17 functions. However, it is interesting to note that unlike mucosal candidiasis, disseminated candidiasis is remarkably uncommon in HIV-infected subjects. The mechanisms are not entirely clear but likely due to the relative normal functions of neutrophils in HIV-infected individuals [54]. Neutrophil activation requires IL-17 signaling and recent evidence has suggested that innate lymphoid cells (ILC) represent an important source of IL-17 to support neutrophil activation in the absence of Th17 CD4 T cells [55], which may help explain why disseminated candidiasis remains uncommon even in HIV-infected patients with severe CD4 T cell depletion.

Mechanisms for *in vivo* depletion of CD4 T cells in HIV-infected subjects might be highly complex. However, direct HIV infection and ongoing viral replication is thought to be a major driving factor for CD4 depletion at both acute and chronic stages of the disease [13, 56, 57]. The *in vitro* system established in our group provides a method to examine differential HIV susceptibility of antigen-specific CD4 T cells and the associated functional profile (Fig A in S1 Appendix). Based on this system, we have demonstrated that *C*. *albicans*-specific CD4 T cells are substantially more susceptible to HIV than CMV-specific CD4 T cells *in vitro* (Fig 2A) [12]. In the current study, we further investigated HIV susceptibility of these two Ag-specific CD4 T-cell populations *in vivo*. Like previously reported [13, 40], we used quantitative PCR to quantify cell-associated HIV load in sorted Ag-specific CD4 cells as an indication of their natural infection history. Ag-specific CD4 T cells were sorted from PBMCs based on CFSE-low, which provided an advantage in that we could obtain Ag-specific cells at relatively higher numbers for subsequent PCR. We showed that peripheral *C*. *albicans*-specific CD4 T cells harbored modestly but significantly higher levels of HIV DNA than CMV-specific CD4 T cells in HIV-infected individuals (Fig 4B). We noted that the differences for HIV load between *C*. *albicans*- and CMV-specific CD4 T cells in some subjects were modest. Considering that lymphoid and mucosal tissues such as GI tract are major sites of HIV infection, we speculate that differences might be more profound for Ag-specific CD4 T cells isolated from lymphoid or mucosal effector sites.

Previous studies have investigated *in vivo* HIV susceptibility of CD4 T cells specific for other important antigens such as MTB and HIV, in addition to CMV [13, 40]. We here included additional antigens for comparison wit *C*. *albicans* (Fig 4C). Since the HIV-infected subjects in RV21 demonstrated very low response to MTB, we were unable to directly compare between MTB and *C*. *albicans* in these subjects. Instead, we compared *C*. *albicans* antigen with varicella zoster virus (VZV) and HIV Env as non-CMV antigen controls. Based on the subjects investigated, we found that *C*. *albicans*-specific CD4 T cells also harbored higher levels of HIV DNA than these two groups of antigen-specific CD4 T cells (Fig 4C), suggesting that the higher HIV susceptibility for *C*. *albicans*-specific CD4 T cells may not be simply due to enhanced protection of CMV-specific CD4 T cells. An important previous study [40] reported that HIV-specific CD4 T cells are highly susceptible to HIV *in vivo*, where CD4 T cells specific for all HIV antigens (gag, env, nef, etc) were measured together. We here also showed that HIV Env-specific cells are susceptible to HIV, albeit at lower levels than *C*. *albicans*-specific CD4 T cells, which might attribute to variations between different cohorts. In addition, it is possible that HIV susceptibility of CD4 T cell subsets specific to different HIV antigens (e.g. Env vs. Gag or Nef) may also significantly vary, which is under investigation in our group. Nevertheless, our results altogether suggest that *C*. *albicans*-specific CD4 T cells are highly susceptible to HIV both *in vitro* and *in vivo*, which may contribute to their rapid depletion during HIV infection.

Phenotypic and functional characteristics of CD4 T cells are shown to be closely associated with their susceptibility to HIV infection, such as expression of IL-2/CD25 [10, 13], MIP-1β [13, 58, 59] and IL-17 [60, 61]. Based on the *in vitro* system (Fig A in S1 Appendix), we showed that compared to MIP-1β or IFN-γ, the IL-17, IL-22 or IL-2-producing subsets of *C*. *albicans*-specific CD4 T cells are more permissive to HIV (Fig 2). We noted that although total IFN-γ+ CD4 T cells are also fairly susceptible to HIV, single IFN-γ+ CD4 T cells in the absence of other cytokine expression (IL-17, IL-2 or IL-22) are substantially more resistant to HIV (Fig 2C), which is consistent with and possibly provides an explanation for earlier reports that IFN-γ+IL-2+ double-producing CD4 T cells are preferentially lost, while the IFN-γ single-producing CD4 T cells are frequently detected in HIV non-controllers [62]. While definitive molecular mechanisms for high HIV susceptibility of *C*. *albicans*-specific CD4 T cells remain not fully clear, data from the current study and our previous reports have suggested that multiple layers of mechanisms may contribute to this observation: 1) less protection of *C*. *albicans*-specific CD4 T cells from HIV at entry level due to limited production of beta-chemokines (Fig F in S1 Appendix and [12]); 2) permissive post-entry environment that favors productive HIV replication, including low expression of antiviral restriction factors [12] and high expression of pro-inflammatory cytokines such as IL-17, IL-22, and IL-2 (Fig 2B and 2C); 3) higher expression of important mucosal homing receptors (α4β7 and CCR6) that enhances exposure of *C*. *albicans*-specific CD4 T cells to HIV at mucosal sites (Fig 5). These multiple layers of mechanisms may act together to render *C*. *albicans*-specific CD4 T cells susceptible to HIV infection and depletion. α4β7 is a key gut mucosal homing receptor that can directly interact with HIV gp120 [45]. It has been suggested that strong α4β7 reactivity may provide an increased fitness for mucosal HIV transmission [45, 46]. Our results showed that expression of α4β7 does not appear to directly correlate with HIV infectivity in CD4 T cells (Fig 5D), which is further supported by the α4β7 blocking experiment (Fig 5E). In agreement with this result, further analysis also showed no difference in cytokine expression (IL-17, IL-2, IFN-γ and MIP-1β) between α4β7+ and α4β7- subsets of *C*. *albicans*-specific CD4 T cells (Fig 5C). These results imply that unlike CD4 and CCR5, α4β7 may not be critically important for *in vitro* HIV replication in our system, where both cells and virus were present at high concentrations and HIV may be able to efficiently bind to CD4 and CCR5 even in the absence of α4β7. This is consistent with some previous reports [48] and results from other groups (personal communication). However, impact of α4β7 on HIV susceptibility of Ag-specific CD4 T cells *in vivo* deserves further investigation.

Mechanisms for how expression of cytokines is associated with HIV susceptibility for Ag-specific CD4 T cells are not fully known. We examined CCR5 expression on different functional CD4 subsets and found no significant difference (Fig E in S1 Appendix); instead, we found that IL-2+, IL-22+ or IL-17+ CD4 T cells express lower levels of MIP-1β compared to IFN-γ+ CD4 T cells (Fig 3A), which might explain the differential HIV infectivity between these cytokine-producing CD4 subsets. This is consistent with an earlier study showing that *in vitro* differentiated IL-17-producing CD4 T cells express comparable levels of CCR5 with IFN-γ-producing CD4 T cells, but are more susceptible to HIV due to lack of beta-chemokine production [38]. Another potentially important correlating factor for HIV susceptibility in different cytokine+ Ag-specific CD4 T cell subsets is CD25. In this study, we showed that productive HIV infection was predominantly observed in CD25+ Ag-specific CD4 T cells and that CD25 co-expressed with IL-2, IL-22 or IL-17, but not IFN-γ+ or MIP-1β (Fig 2E–2G). Lastly, post-entry mechanisms might also be involved in regulating HIV susceptibility in different cytokine-producing CD4 T cells. For instance, polarized Th1-like, IFN-γ-producing CD4 T cells, such as CMV-specific CD4 T cells, which can acquire direct antiviral functions [58], were shown to be able to activate a broad array of innate antiviral factors and manifest strong post-entry inhibition of HIV replication [12]. Molecular mechanisms for how different cytokine signaling may affect HIV infectivity in target CD4 T cells remain largely unknown and further investigation is warranted.

In summary, in the present study, based on an ART naïve HIV infection cohort, we comparatively investigated the longitudinal impact of HIV on *C*. *albicans*- and CMV-specific CD4 T-cell immunity in HIV non-controllers. We identified a sequential dysfunction and preferential depletion of *C*. *albicans*-specific CD4 T cell response during progressive HIV infection. These findings may provide an immunological basis for early loss of immune control over mucosal candidiasis in HIV-infected individuals and also suggest a potential mechanism for pathogen-specific immune failure in AIDS.

## Materials and Methods

### Ethics statement

The study involves use of PBMC samples from healthy human donors as well as from HIV-infected subjects enrolled in RV21 cohort, an ART naïve longitudinal HIV infection cohort established by US MHRP. Healthy donor PBMCs were obtained from University of Texas Medical Branch (UTMB) blood bank and the RV21 PBMC samples were obtained from MHRP. Characteristics of HIV-infected subjects were summarized in Table 1. All samples were analyzed anonymously and investigators of this study have no access to any subject identification information. The study was determined as non-human subject research and approved by both UTMB and MHRP IRBs. Written informed consents were obtained from study participants.

### Antigens and HIV virus

Antigens used for long-term (6 days) or short-term (overnight) stimulation of PBMCs include: *C*. *albicans* extracts (Greer Laboratories), *C*. *albicans* MP65 peptides (JPT), varicella zoster virus lysates (Advanced Biotechnologies), CMV lysates (Advanced Biotechnologies), CMV pp65 peptides and HIV Env peptides (NIH AIDS reagent program). HIV-1 US1 (GS004), an R5 subtype B isolate, was obtained through the NIH AIDS reagent program and used for *in vitro* HIV infection.

### CFSE labeling and antigen stimulation of PBMC

CFSE labeling and antigen stimulation of PBMCs were performed as previously described [12, 34]. Briefly, PBMCs were washed twice with staining buffer (RPMI-1640 medium containing 1% FBS) (Life Technologies, USA), followed by labeling with 1.0 μM CFSE (Life Technologies, USA) at a cell concentration of 2×10^7^ cells/ml for 8 minutes at room temperature (RT) in dark. Equal volume of pre-warmed FBS was then added to cells for incubation at RT for 4 minutes to quench CFSE. CFSE-labeled PBMCs were subjected to antigen stimulation and various subsequent assays. For antigen stimulation, cells were first pulsed with whole antigens (*C*. *albicans*: 1:200; CMV: 5 μg/ml) at high cell concentrations (1×10^7^ cells/ml) for 3–4 hours in tubes, and then diluted to 2×10^6^ cells/ml for normal cell culture in culture plate for several days to stimulate antigen-specific T cell activation and proliferation. For experiments involving cell sorting and HIV DNA quantification for PBMCs of HIV-infected subjects (RV21), cells were also stimulated with VZV antigen (final concentration: 5μg/ml) and HIV Env peptides (final concentration: 1μg/ml). Different assays were subsequently performed as detailed below.

For normal PBMCs, stimulated cells were cultured for ~6 days and the proliferating CD4 T cells, identified by CFSE dilution (CFSE-low), in stimulated PBMCs were subjected to multiple analyses: 1) cytokine expression by flow cytometer; 2) Ag-specific CD4 cell sorting using FACS Aria for subsequent gene-expression analysis. In addition, the Ag-stimulated normal PBMCs were also subjected to *in vitro* HIV infection (day 3 after initial Ag stimulation) for the examining the HIV susceptibility of Ag-specific CD4 T cells. For PBMCs from HIV-infected subjects, CFSE-labeled and antigen-stimulated cells were cultured for ~6 days and subjected to: 1) analysis for cytokine expression and proliferative response (CFSE-low); 2) Ag-specific CD4 cell sorting for the *in vivo* HIV infectivity analysis. Methods for each assay were detailed below.

### *In vitro* HIV infection

Three days after CFSE labeling and initial antigen stimulation, PBMCs of healthy donors were infected with pre-titrated HIV R5 (US1; 50ng/ml p24). In some experiments, prior to HIV infection, antigen-stimulated cells were pre-incubated with anti-human α4β7 antibody (ACT-1) (5 μg/ml) to block the interaction between HIV (envelope protein) and α4β7 present on antigen-specific CD4 T cells. Twenty-four hours after HIV exposure, free HIV virions were washed away from the cell culture. The infection was maintained for additional 2 days and cells were re-stimulated with PMA (500 ng/ml) and ionomycin (1μg/m) for *de novo* cytokine synthesis (3 days after HIV exposure). Productive HIV infection of antigen-specific CD4 T cells in PBMCs and the associated functional (cytokine) or phenotypic parameters were examined by multi-color flow cytometry based on intracellular HIV p24 expression in CFSE-low proliferating CD4 T cells as previously described [12, 34].

### Short-term *ex vivo* antigen stimulation of PBMCs from HIV-infected subjects

In addition to 6-day stimulation, PBMCs from RV21 HIV-infected subjects (early and chronic time points) were also stimulated with *C*. *albicans* and CMV antigens for overnight in the presence of anti-CD28/CD49d antibody cocktail and protein transport inhibitors (BD Bioscience). For short-term stimulation, *C*. *albicans* MP65 and CMV pp65 peptides were used. Intracellular cytokine staining and flow cytometric analysis were performed, as detailed below, to measure de nova frequencies of antigen-specific CD4 T cells in PBMCs of HIV-infected subjects.

### Cell staining and flow cytometric analysis

#### 6-day stimulation of CFSE-labeled PBMCs

6-day stimulated, CFSE-labeled PBMCs were subjected to three different panels of staining to measure: phenotypic and cytokine profile, HIV infectivity (p24) and intracellular transcription factors (T-bet and EOMES) in CFSE-low, Ag-specific CD4 T cells. Briefly, cells were first stained for viability with LIVE/DEAD Fixable Aqua Blue (Life Technologies), followed by surface makers staining, including CD3-APC-H7 (BD Bioscience), CD4-PE-Cy5 (BD Bioscience), CD8-BV785 (Biolegend), CD25-PE-Cy5 (Biolegend), CCR5-Pacific Blue (BD Bioscience), α4β7-APC (NIH AIDS Reagent Program), CCR6-APC (R&D Systems), CCR9-PerCP-Cy5.5 (Biolegend). After surface staining, cells were fixed, permeabilized (BD Bioscience) and subjected to intracellular staining, including HIV p24-PE (Beckman Coulter), IL-17-Alexa Fluor 488 (eBioscience), IL-22-APC (eBioscience), IL-2-PerCP-Cy5.5 (Biolegend), IFN-γ-Alexa Fluor 700 (eBioscience), MIP-1β-PE-Cy7 (BD Bioscience). Combination of surface and intracellular staining antibodies may vary according to different assays. For intracellular staining for T-bet and EOMES, cells were fixed and permeabilized using eBioscience’s transcription factor staining buffer set (eBioscience) according to manufacturer’s instructions. Cells were then subjected to intracellular staining with anti-T-bet-Pacific Blue (Biolegend) and anti-EOMES PE-eFluor 610 (eBioscience).

#### Short-term ex vivo stimulated PBMC

Following overnight stimulation as described above, PBMCs (RV21 HIV-infected subjects) were subjected to staining and flow cytometric analysis for measuring de nova frequencies of *C*. *albicans* and CMV-specific CD4 T cells. Cells were first stained for viability with LIVE/DEAD Fixable Aqua Blue (Life Technologies), followed by surface staining, including CD3-APC-H7 (BD Bioscience), CD4-PE-Cy5 (BD Bioscience), CD8-BV785 (Biolegend). Cells were then fixed, permeabilized (BD Bioscience) and subjected to intracellular staining, including IL-17-Alexa Fluor 488 (eBioscience), IL-22-APC (eBioscience), IL-2-PerCP-Cy5.5 (Biolegend), IFN-γ-Alexa Fluor 700 (eBioscience), MIP-1β-PE-Cy7 (BD Bioscience). Following staining, cells were acquired using the LSRII Fortessa Analyzer (BD) and data were analyzed using FlowJo (Tree Star). Where appropriate, Boolean gating and Spice analysis were performed using the FlowJo program to analyze the poly-functional characteristics of antigen-specific CD4 T cells.

### Antigen-specific CD4 T-cell sorting

Sorting antigen-specific CD4 T cells from PBMCs was performed either for quantification of cell-associated HIV DNA (HIV-infected subjects in RV21) or for gene-expression analysis (healthy PBMC). CFSE-labeled, Ag-stimulated PBMCs were first stained with Fixable LIVE/DEAD Violet Dead Stain Kit (Life Technologies), followed by staining for surface markers including CD3-APC-H7, CD4-PE-Cy5 and CD8-PE (BD Bioscience). Cells (from HIV+ subjects) were then fixed and subjected to sorting of antigen-specific CD4 T cells, based on CFSE-low CD3+ CD8-, as well as the CFSE-Hi non-specific CD4 T cells using FACS Aria (BD). Sorted cells were subsequently subjected to quantification of HIV DNA (below). Cells from healthy donors were live sorted for antigen-specific CD4 T cells, followed by RNA extraction and gene-expression analysis (below).

### RNA extraction and gene-expression analysis

Total RNA was extracted from live-sorted, antigen-specific CD4 T cells using Quick-RNA MicroPrep kit (Zymo) according to the manufacturer's protocol. Gene expression was quantified using iTaq Universal SYBR Green Supermix (Bio-rad) and the CFX Connect Real-Time PCR Detection System (Bio-rad) after reverse transcription from RNA into cDNA using iScript Reverse Transcription Supermix for RT-qPCR (Bio-rad). Primers were designed to amplify the target genes (human T-bet, EOMES and RORC). Primer sequences for gene expression analysis were shown in Table 2. The relative quantity of gene expression was calculated using the 2^-ΔΔCt^ method.

**Table 2:** Primer sequences for quantification of gene expression.

Gene	Gene Bank #	Sequence
GAPDH	NM_001256799.2	F: CAATGACCCCTTCATTGACC
		R: GACAAGCTTCCCGTTCTCAG
CCL-20	NM_004591.2	F: GTGGCTTTTCTGGAATGGAA
		R: CAAGTCCAGTGAGGCACAAA
CCL-25	NM_001201359.1	F: GATAAAACCGTCGCCCTACA
		R: ATCAGGCCAACTCCCTCTTT
EMOES	NM_001278182.1	F: GGCAAAGCCGACAATAACAT
		R: TTCCCGAATGAAATCTCCTG
T-bet	NM_013351.1	F: GGTTGGAGGACACCGACTAA
		R: ATCCTTCTTGAGCCCCACTT
RORC	NM_001001523.1	F: CAGGCTTTATGGAGCTCTGC
		R: TCCTAACCAGCACCACTTCC

### Quantification of cell-associated HIV DNA

Genomic DNA was extracted from the fixed, sorted antigen-specific CD4 T cells in PBMCs of HIV+ subjects. After washing once in PBS, sorted cells were lysed in lysis buffer (10mM Tris, 5mM EDTA, 1% SDS pH8.0) for 1 hour at room temperature and then digested with 32 U/ml of Protease K (New England Biolabs) for 2 hours at 56°C. After Protease K inactivation at 95°C for 30 min, genomic DNA was purified and solved in Tris-Cl (10 mM, PH 8.0). HIV DNA was quantified using iTaq Universal SYBR Green Supermix (Bio-rad) and the CFX Connect Real-Time PCR Detection System (Bio-rad) according to the manufacturer's protocol. Primers used to amplify HIV Gag and the control GAPDH genes were shown in Table 3. pNL4-3 and recombinant plasmid encoding GAPDH gene were used to generate standard curves (Fig I in S1 Appendix). The absolute quantity of HIV DNA copies was calculated based on standard curves.

**Table 3:** Primer sequences of HIV DNA quantification.

Gene	GeneBank#	Sequence
GAPDH	NG_007073.2	F: GGCCTCCAAGGAGTAAGACC
		R: AGGGGTCTACATGGCAACTG
HIV Gag	AF324493.2	F: GGAAGCTGCAGAATGGGATA
		R: GCTATGTCACTTCCCCTTGG

### Statistical analysis

Statistical analysis was performed using Prism 6.0 (GraphPad). Statistical comparison between groups was performed using paired or non-paired t test. Two-tailed p values were denoted, and p values < 0.05 were considered significant.

## Supporting Information

S1 Appendix Supplementary Figures (A-I) and the corresponding figure legends are included in the S1 Appendix. (DOCX)
