# Supplementary material for: Sequential Dysfunction and Progressive Depletion of Candida albicans-Specific CD4 T Cell Response in HIV-1 Infection
Source: PLoS Pathog. 2016 Jun 9;12(6):e1005663. doi: 10.1371/journal.ppat.1005663 (PMC4900544; doi:10.1371/journal.ppat.1005663)
Supplement: S1 Appendix — (DOCX) [file ppat.1005663.s001.docx]

**SUPPLEMENTARY MATERIALS**

**Sequential dysfunction and progressive depletion of *Candida albicans*-specific CD4 T cell response in HIV-1 infection**

Fengliang Liu^1^, Xiuzhen Fan^1^, Sarah Auclair^1^, Monique Ferguson^2^, Jiaren Sun^1^, Lynn Soong^1^, Wei Hou^3^, Robert Redfield^4^, Deborah Birx^5^, Silvia Ratto-Kim^6^, Merlin Robb^6^, Jerome Kim^7^, Nelson Michael^5^, Haitao Hu^1^**^*^**

**Figure A**

**Figure A. Summary of the system.** PBMCs of human subjects who manifest positive responses to antigens of interest (e.g. *C. albicans* and CMV) were labeled with CFSE, a fluorescent dye to track cell division, and then stimulated with *C. albicans* or CMV antigen, followed by exposure to HIV for infection on day 3 post antigen stimulation. (**A**) After additional 3 days, HIV infection of Ag-specific CD4 T cells in PBMC and the associated phenotypic or functional characteristics were examined by multi-color flow cytometry based on intracellular p24 content in CFSE-low CD4 T cells. Representative flow cytometry data are shown for intracellular p24 expression in CFSE-low CD4 T cells with or without HIV exposure. In some assays, in order to simultaneously examine functional characteristics of Ag-specific CD4 T cells in addition to HIV p24, cells were briefly stimulated with PMA and ionomycin for *de novo* cytokine synthesis. (**B**) Different CFSE-low Ag-specific CD4 T-cell populations from the same PBMC can also be sorted for gene-expression analyses or cell-associated HIV DNA quantification.

**Figure B**


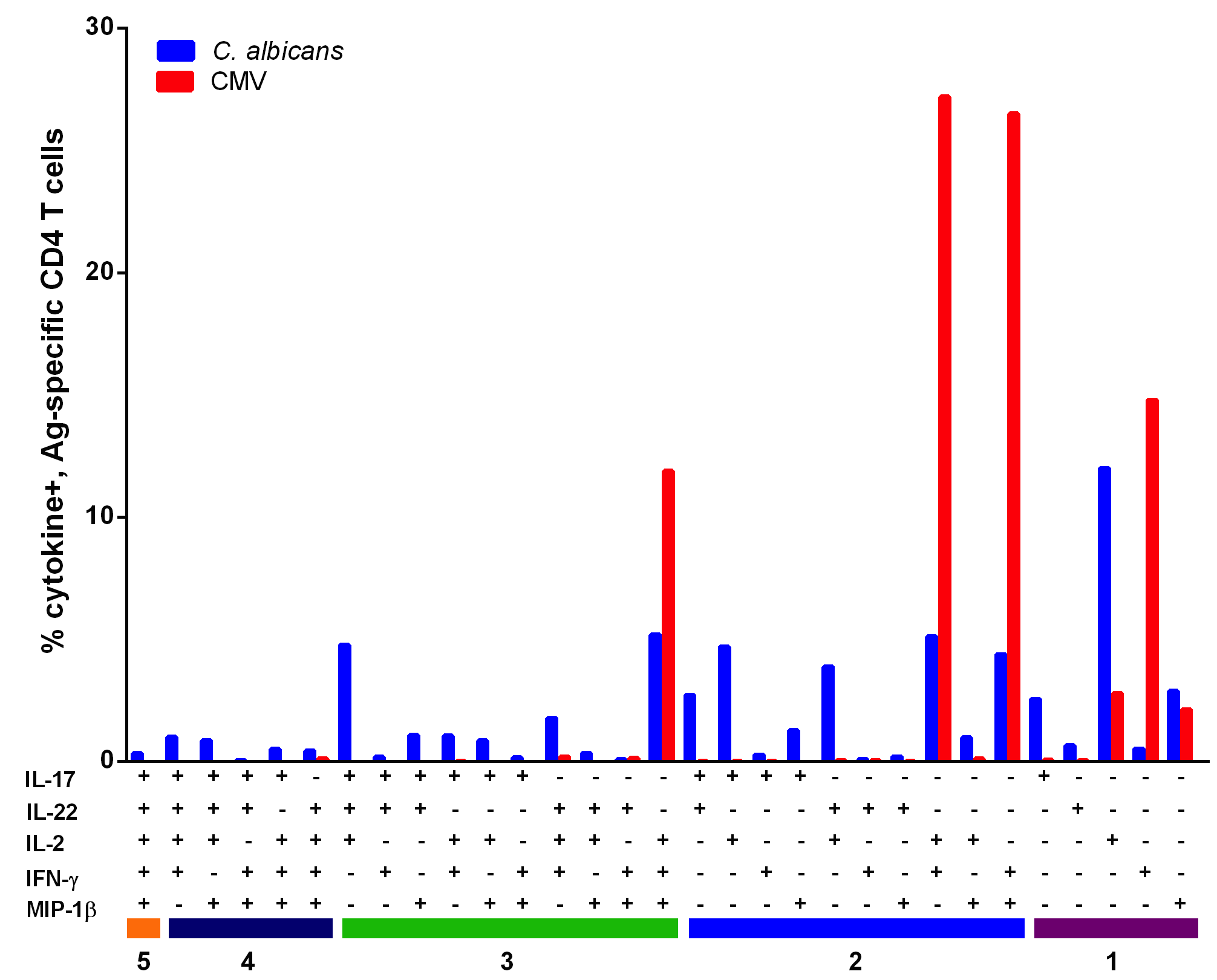


**Figure B. Poly-functional analysis of *C. albicans*- and CMV-specific CD4 T cell response in PBMC of healthy subject.** Poly-functional profiles (expression of combination of cytokines IL-17, IL-22, IL-2, IFN-γ and MIP-1β) of CFSE-low, *C. albicans*- (blue) and CMV- (red) specific CD4 T cells were analyzed using Boolean gating analysis. The x-axis displays all different functional subsets and the y-axis shows percentage of individual functional subsets within the CFSE-low, Ag-specific CD4 T-cell population. The numbers below color-coded bar show number of cytokines expressed by the functional subsets.

**Figure C**

**
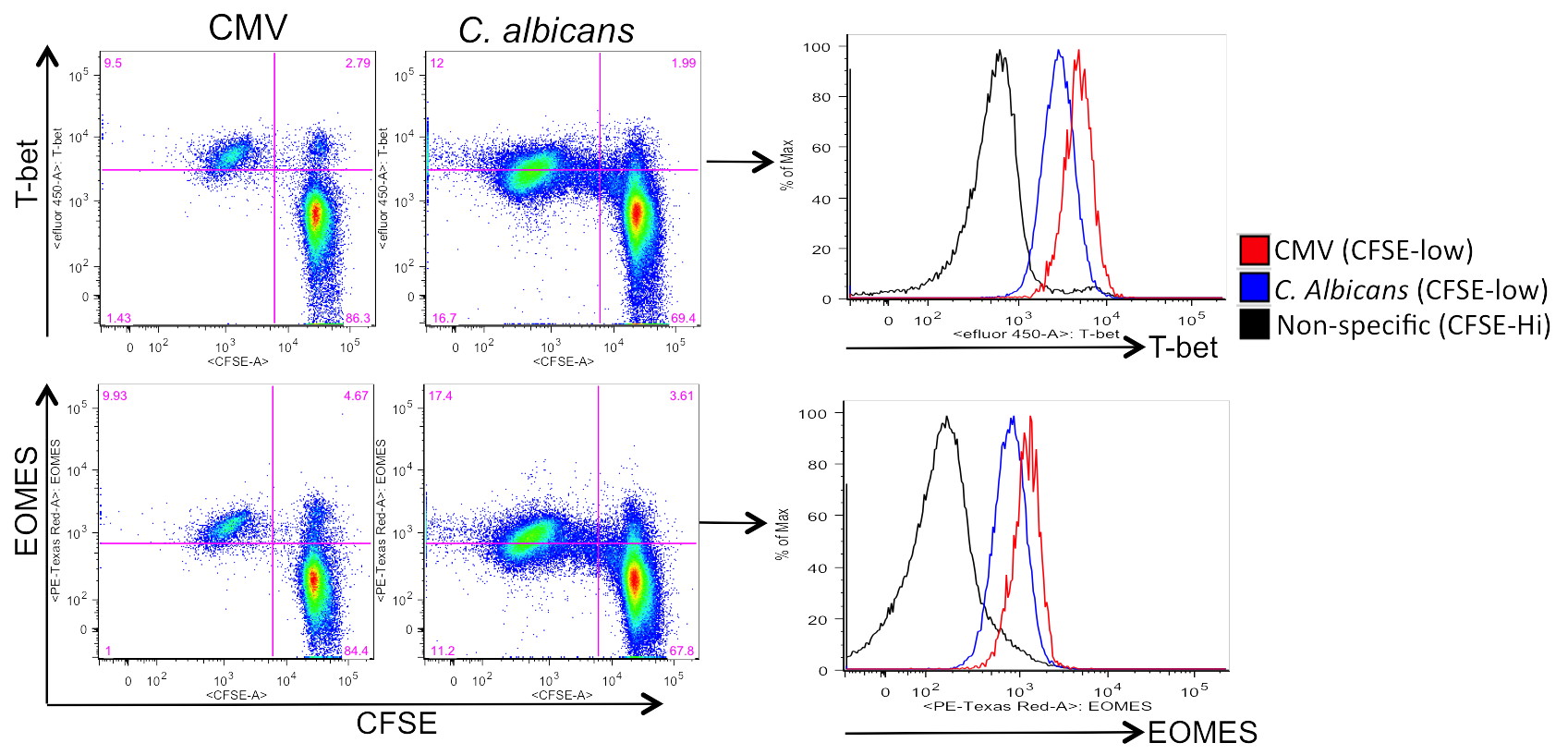
**

**Figure C. Flow cytometric analysis of T-bet and EOMES expression in CFSE-low and CFSE-hi CD4 T cells in PBMC after antigen stimulation.** PBMC of healthy donors were CFSE-labeled and stimulated with Ag (*C. albicans* and CMV) as described in the method summary (Fig. S1). Intracellular expression of T-bet (top) and EOMES (bottom) in CFSE-low, Ag-specific CD4 T cells as well as in CFSE-Hi CD4 T cells were analyzed by flow cytometry. Both flow cytometric dot plots (left panels) and histograms (right) are shown for comparison.

**Figure D**


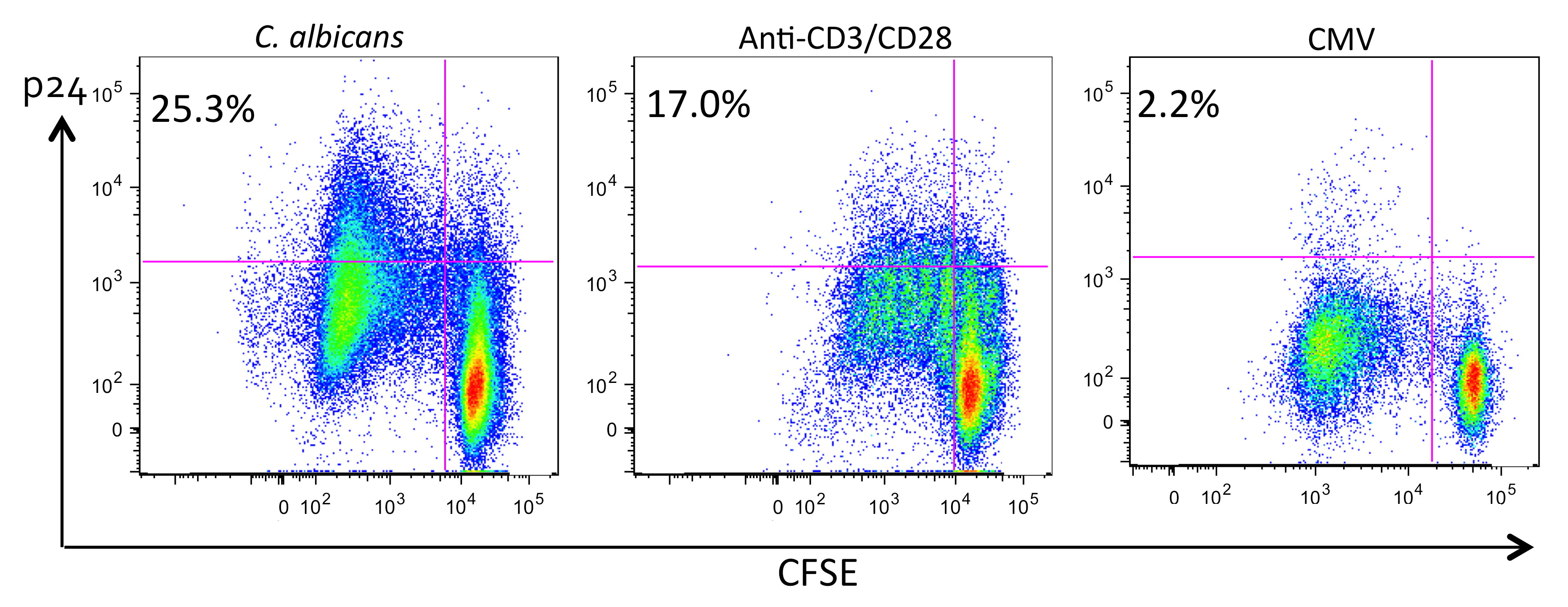


**Figure D. In vitro HIV susceptibility of *C. albicans-* and CMV-specific CD4 T cells as compared to globally activated total CD4 T cells.** CFSE-labeled PBMCs were stimulated with *C. albicans* (left), anti-human CD3/CD28 (middle), or CMV (right) for 3 days, and exposed to HIV for infection. Productive HIV infection of Ag-specific or globally activated CD4 T cells in PBMC was examined as intracellular p24+ rates in CFSE-low CD4 T cells (number in top-left quadrant).

**Figure E**

**Figure E.** **CCR5 expression on different functional subsets of *C. albicans*-specific CD4 T cells.** Expression of CCR5 on each cytokine-producing subset of CFSE-low *C. albicans*-specific CD4 T cells was determined by flow cytometry (mean fluorescence intensity) and compared between different functional subsets.

**Figure F**

**Figure F.** **Expression of MIP-1β in IL-22, IL-17 and IL-2 functional subsets of *C. albicans*-specific CD4 T cells as compared to IFN-γ subset.** Flow cytometry histogram comparison for expression of MIP-1β between IL-22, IL-17, IL-2 and IFN-γ functional subsets of *C. albicans*-specific CD4 T cells.

**Figure G**

**Figure G. Co-expression of IL-2, IL-17 and IL-22 with IFN-γ or MIP-1β in *C. albicans*-specific CD4 T-cell population.** CFSE-low, total IFN-γ-producing (top) or total MIP-1β-producing (bottom) *C. albicans*-specific CD4 T cells were gated**.** Co-expression of IFN-γ or MIP-1β with other cytokines (IL-2 and IL-17 or IL-22) was analyzed. High fractions of IFN-γ+ cells also co-express IL-2 (~63.2%), IL-17 (13.2%) or IL-22 (15.2%); significant fractions of MIP-1β cells co-express IL-2 (~26.6%), IL-17 (12.1%) or IL-22 (8.4%).

**Figure H**

**Figure H. Detection of HIV in supernatant of PBMC from HIV-infected subjects stimulated with different antigens in the presence of AZT.** PBMC were stimulated with *C. albicans*, CMV, VZV, or HIV Env in the presence of AZT to inhibit de novo viral replication. On day 5 supernatant was collected to measure HIV using the luciferase-based TZM-bl infection method. The readout (relative light units) was compared with negative control (medium only) and positive control (supernatant of in vitro HIV-infected normal PBMC). The data showed that no HIV was detected from RV21 PBMC when stimulated with antigens in the presence of AZT.

**Figure I**

**
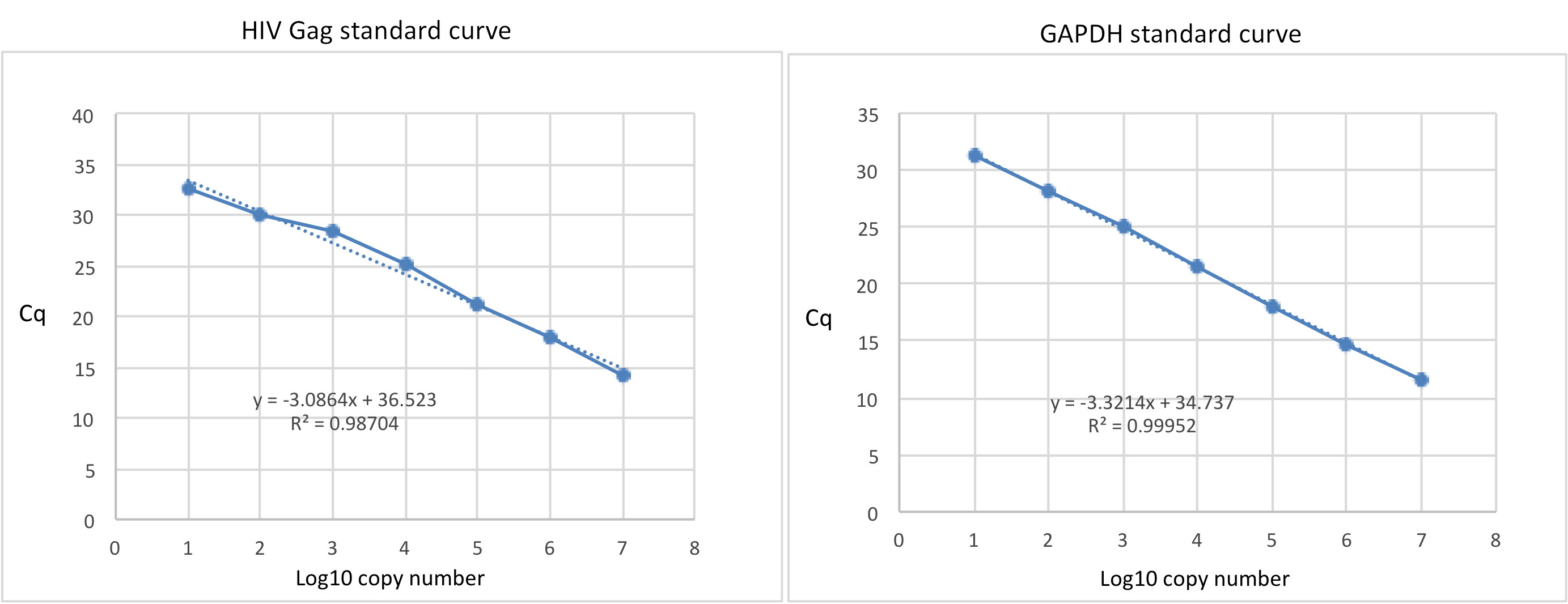
**

**Figure I. Generation of standard curves for HIV Gag and cellular GAPDH.** The HIV Gag plasmid pNL4-3 and plasmid encoding GAPDH (pGEM-T-GAPDH) were used as templates in quantitative PCR to generate standard curves. Serially diluted plasmids (10^1^, 10^2^, 10^3^, 10^4^, 10^5^, 10^6^ or 10^7^ copies) were added to qPCR reaction and the generated quantification cycle (Cq) values (y axis) and log10 copy numbers (x-axis) were used to create standard curves.
